# Impairment Mechanisms and Intervention Approaches for Aged Human Neuromuscular Junctions

**DOI:** 10.3389/fnmol.2020.568426

**Published:** 2020-11-16

**Authors:** Yomna Badawi, Hiroshi Nishimune

**Affiliations:** ^1^Department of Anatomy and Cell Biology, University of Kansas School of Medicine, Kansas City, KS, United States; ^2^Neurobiology of Aging, Tokyo Metropolitan Institute of Gerontology, Itabashi, Japan

**Keywords:** active zone (AZ), aging, caloric restriction, exercise, laminin, neuromuscular disease, progeria, synapse

## Abstract

The neuromuscular junction (NMJ) is a chemical synapse formed between a presynaptic motor neuron and a postsynaptic muscle cell. NMJs in most vertebrate species share many essential features; however, some differences distinguish human NMJs from others. This review will describe the pre- and postsynaptic structures of human NMJs and compare them to NMJs of laboratory animals. We will focus on age-dependent declines in function and changes in the structure of human NMJs. Furthermore, we will describe insights into the aging process revealed from mouse models of accelerated aging. In addition, we will compare aging phenotypes to other human pathologies that cause impairments of pre- and postsynaptic structures at NMJs. Finally, we will discuss potential intervention approaches for attenuating age-related NMJ dysfunction and sarcopenia in humans.

## Introduction

Synapses are required to maintain the proper function of the nervous system both in health and during disease. The neuromuscular junction (NMJ) is a synapse critical for the transfer of information between a presynaptic motor neuron and a postsynaptic muscle fiber at specialized sites on the muscle plasma membrane called endplates. During development, motor neurons seek out and find nascent endplates on the sarcolemmal surface (skeletal muscle fiber plasma membrane) (Frank and Fischbach, 1979; Sanes and Lichtman, 2001). Innervation of muscle fibers by motor neurons ensures proper control of skeletal muscle contraction through the regulated release of neurotransmitters. Among vertebrate NMJs, the neurotransmitter acetylcholine (ACh) is released from highly specialized sites at the presynaptic membrane called active zones. By electron microscopy analysis, active zones appear as electron-dense projections on the presynaptic membrane where synaptic vesicles fuse for subsequent exocytosis (Couteaux and Pecot-Dechavassine, 1970; Tsuji, 2006). In human NMJs, freeze-fracture electron microscopy shows double parallel rows of about 20 large particles in the p-face of the presynaptic membrane, which was interpreted as an active zone unit structure (Fukunaga et al., 1982). The macromolecules in the active zones interact with docked synaptic vesicles, which has been revealed in frog and mouse NMJs by electron microscopy tomography (Harlow et al., 2001; Nagwaney et al., 2009; Szule et al., 2012; Harlow et al., 2013; Jung et al., 2018). These docked synaptic vesicle fuse with the plasma membrane following calcium influx into the presynaptic terminals with the help of SNARE (Soluble *N*-ethylmaleimide-sensitive factor activating protein receptor) proteins including SNAP-25, synaptobrevin/VAMP, and syntaxin 1 (Jahn and Scheller, 2006; Sudhof and Rothman, 2009). The calcium influx required for neuromuscular transmission at adult mammalian NMJs is primarily mediated by P/Q-type voltage-gated calcium channels (VGCC) (Uchitel et al., 1992; Rosato Siri and Uchitel, 1999). Electron microscopy studies have shown that presynaptic active zones are positioned apposed to the openings of post-synaptic junctional folds (Couteaux and Pecot-Dechavassine, 1970; Patton et al., 2001). Junctional folds are small invaginations of the postsynaptic membrane that increases the area of acetylcholine receptors (AChRs) accumulation to efficiently receive ACh released from motor nerve terminals following depolarization by the action potential (Fertuck and Salpeter, 1974, 1976; Anderson and Cohen, 1977; Frank and Fischbach, 1979). Voltage-gated sodium channels are located in the troughs of post-synaptic junctional folds to generate action potentials (Caldwell, 2000). The activation of voltage-gated sodium channels in mice is responsible for 15–20% of the endplate potential (Hong and Chang, 1989). The high density of voltage-gated sodium channels and the increased local input resistance due to the narrow structure at the junctional folds are expected to amplify the neurotransmission for more efficient muscle depolarization (Wood and Slater, 2001). For the organization of postsynaptic specialization, agrin (a proteoglycan) is released from nerve terminals and binds to the low-density lipoprotein receptor (LDLR)-related protein 4 (LRP4) localized in the postsynaptic membrane (DeChiara et al., 1996; Kim et al., 2008). The interaction of agrin and LRP4 activates the protein tyrosine kinase function of the co-receptor muscle-specific kinase (MuSK), which localize together with AChR (Valenzuela et al., 1995). The activation of MuSK is essential for the clustering of AChRs through the AChR-associated protein Rapsyn (Apel et al., 1997; Lee et al., 2009) and the organizer protein Dok-7 (Beeson et al., 2006; Okada et al., 2006; Hallock et al., 2010).

**FIGURE 1:**
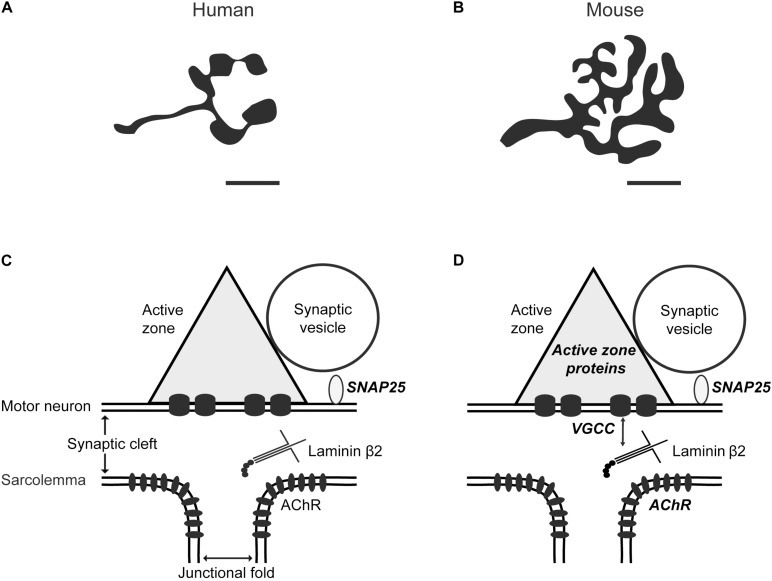
Comparison between human and mouse neuromuscular junctions. Schematic diagrams show **(A)** human versus **(B)** mouse NMJs based on nerve-specific stains (adapted from Jones et al., 2017). The NMJ size and axon diameter are significantly smaller in humans compared to mice. Scale bar, 10 μm. A diagram depicting an active zone at the **(C)** human and **(D)** mouse NMJs. In mice, muscle-derived synapse organizer laminin β2 interacts with presynaptic voltage-gated calcium channels (VGCC) to organize active zones (Nishimune et al., 2004). Super-resolution microscopy revealed the nanoscale localization of the SNARE protein synaptosomal associated protein-25 (SNAP25) in human and mouse NMJs (Jones et al., 2017), active zone-specific proteins, and acetylcholine receptors (AChR) in mice (Nishimune et al., 2016; York and Zheng, 2017), which are described in bold italic characters. The nanoscale localization of active zone-specific proteins and acetylcholine receptors in human NMJs has not been revealed yet.

Impairments of active zones are seen in pathological conditions, including the autoimmune disease, Lambert–Eaton myasthenia syndrome (LEMS), and Pierson’s syndrome. LEMS is caused by autoantibodies including ones against P/Q-type VGCCs, and Pierson syndrome occurs due to mutation in *lamb2* gene (Takamori et al., 2000; Zenker et al., 2004). Active zone loss may occur in these diseases due to a disruption in the interaction between muscle-derived laminin β2 (Sanes and Hall, 1979; Hunter et al., 1989) and P/Q-type VGCC, which is necessary for the active zone organization (Figure 1) (Nishimune et al., 2004).

Although NMJs in most vertebrate species share many essential features, there are also significant differences (Figure 1), which will be described in this review. Human NMJs, are among the smallest and release the smallest number of ACh containing synaptic vesicles at the presynaptic terminal (Oda, 1984; Slater et al., 1992; Slater, 2015, 2017). However, they have very extensive postsynaptic junctional folds, which may amplify transmitter action (Wokke et al., 1990; Slater et al., 1992). Human NMJs are also susceptible to pathological processes and are targets in several neurodegenerative conditions, including aging. This review will focus on recent findings regarding human NMJs and a comparison of what is known about other mammalian NMJs. We will describe age-related changes at human NMJs and insights about potential mechanisms of aging obtained from animal models of accelerated aging. Furthermore, we will compare aging to other human neurological diseases that cause impairments of pre- and post-synaptic structures at NMJs.

## Structure of the Human Neuromuscular Junction

Human NMJs analyzed by freeze-fracture electron microscopy show double parallel rows of large intramembranous particles on the cytosolic half of a plasma membrane (the P-face), containing 3–15 particles per row spaced 20 nm apart (Fukunaga et al., 1982). This structure is considered to represent an active zone unit. Similar structures are identified in rodent and lizard NMJs, which appear as double parallel rows of large intramembranous particles on the P-face (Ellisman et al., 1976; Walrond and Reese, 1985; Fukuoka et al., 1987). In humans, these active zone units are distributed at a density of 2.6 active zones per μm^2^ (Fukunaga et al., 1982). Similarly, active zone units in adult mouse NMJs appear in discrete locations within one presynaptic terminal of NMJs at a density of about 2.3–2.7 active zones per μm^2^ (Fukunaga et al., 1983; Fukuoka et al., 1987; Chen et al., 2012) (Table 1).

**TABLE 1:** Comparison between human and mouse NMJs.

	**Human**	**Mouse**
**Pre-synaptic**		
Active zone size	153 × 96 nm (Fukunaga et al., 1982)	80 × 73 nm (Fukuoka et al., 1987)
Active zone density	2.6 per μm^2^ (Fukunaga et al., 1982)	2.3–2.7 per μm^2^ (Fukunaga et al., 1983; Fukuoka et al., 1987; Chen et al., 2012)
Bassoon and PQ-VGCC puncta density per NMJ	Not reported	5–6 puncta per μm^2^ (Nishimune et al., 2016)
SNAP25 puncta density per NMJ	∼15 puncta per μm^2^ (Jones et al., 2017)	∼15 puncta per μm^2^ (Jones et al., 2017)
Quantal content	20–30 (Slater, 2017)	50–100 (Slater, 2017)
Nerve terminal area	122.7 μm^2^ (Boehm et al., 2020)	304.0 μm^2^ (Boehm et al., 2020)
**Post-synaptic**		
Endplate area	351.5 μm^2^ (Boehm et al., 2020)	678.2 μm^2^ (Boehm et al., 2020)
AChR number per NMJ	1.3–3.4 × 10^7^ (Pestronk et al., 1985; Slater et al., 1992)	3.1–8.7 × 10^7^ (Albuquerque et al., 1974)
Area of AChR clusters	71.7 μm^2^ (Boehm et al., 2020)	238.5 μm^2^ (Boehm et al., 2020)

The application of super-resolution microscopy has revealed distribution patterns of synaptic proteins across different species at unprecedented details (Kittel et al., 2006; Willig et al., 2006; Dani et al., 2010; Ehmann et al., 2014; Nishimune et al., 2016; Jones et al., 2017). The molecular architecture of the human NMJ has been recently investigated using confocal and super-resolution microscopy (Jones et al., 2017). Confocal microscopy of motor nerve terminals at human NMJs showed distinctive “hotspots” of fluorescence of the synaptic vesicle protein SV2. This distribution pattern was qualitatively different from mouse nerve terminals, which showed homogeneity of SV2 labeling (Jones et al., 2017). Additionally, direct Stochastic Optical Reconstruction Microscopy (dSTORM) microscopy has been used to assess the nanoscale distribution of synaptosomal associated protein-25 (SNAP25), a SNARE protein in human NMJs. Parallel imaging of human and mouse NMJs using dSTORM revealed several differences between the two species. The intensity of SNAP25 labeling, the density of SNAP25 puncta within each bouton, the average area of SNAP25 puncta, and the area of SNAP25 puncta as a percentage of the total synaptic bouton area were all significantly greater in human NMJs compared to mouse NMJs (Jones et al., 2017). The total area of SNAP25 labeling per NMJ is similar in both humans and mice at approximately 15 μm^2^ per NMJ (Table 1).

Interestingly, this SNAP25 puncta density (approximately 15 puncta per μm^2^) is very similar to the density of other active zone proteins Bassoon, Piccolo and P/Q-type VGCC (approximately 10 puncta per μm^2^) as revealed in mouse NMJs by Stimulated Emission Depletion (STED) super-resolution microscopy (Nishimune et al., 2016). Jones et al. (2017) reported that they were unable to label human NMJs with antibodies against Bassoon or Piccolo, suggesting possible species-specific differences in these antigens (Jones et al., 2017). Although the distribution pattern of these active zone-specific proteins is unknown in human NMJs (Figure 1), analysis of mouse NMJs revealed punctate and overlapping patterns of active zone proteins Bassoon and P/Q-type VGCCs (Nishimune et al., 2016). Interestingly, active zone protein Piccolo does not co-localize with Bassoon. Instead, Piccolo puncta sandwiched a Bassoon punctum in a side-by-side pattern (Nishimune et al., 2016). Current progress in the understanding of active zone nanoarchitecture and mechanisms underlying active zone organization in mammalian NMJs has been reviewed previously (Badawi and Nishimune, 2018). Future studies using super-resolution microscopy will reveal a more detailed subcellular localization of active zone proteins and address species-specific differences that may exist.

The NMJ size relative to muscle fiber diameter is smaller in humans compared to lower vertebrates (frogs and mice), as revealed by electron microscopy (Slater et al., 1992) (Table 1). Confocal microscopy also revealed that human NMJs have a unique morphology, being significantly smaller and more fragmented compared to mice and rats. The axon diameter is 3.69 fold smaller, and the average area of AChR clusters is 3.33 fold smaller in humans compared to mice (Jones et al., 2017). The average number of AChRs in human vastus lateralis muscle is 2.6 × 10^7^, as determined by bungarotoxin binding sites (Slater et al., 1992). In mice, the average number of AChRs is 8.7 × 10^7^ in the sternomastoid muscle and 3.1 × 10^7^ in the diaphragm, also determined by bungarotoxin binding sites (Albuquerque et al., 1974). In general, AChRs in mice cluster at a concentration of approximately 10,000 per μm^2^ in the postsynaptic membrane (Fertuck and Salpeter, 1976; Salpeter and Loring, 1985). The degree of overlap between the presynaptic motor nerve terminal and the post-synaptic component is similar between humans and mice (Slater et al., 1992; Jones et al., 2017). Although human NMJs are smaller than mouse NMJs, human motor nerves innervate muscle fibers up to twice the diameter of those in mice. The smaller size of human NMJs is not due to increased body size and mass because the NMJ size of mice and rats is similar, although rats are approximately 10-fold larger than mice (Jones et al., 2017). Furthermore, human NMJs have extensive folding of post-synaptic junctional folds, which increases the local area of membrane about eight-fold while the increase is about six-fold for mouse NMJs (Slater et al., 1992; Slater, 2017), possibly resulting in post-synaptic amplification of neurotransmitter action through the presence of voltage-gated sodium channels in high density and the increased local input resistance (Slater, 2017). On the postsynaptic side of the mouse NMJ, dSTORM revealed the nanoscale distribution of AChRs (York and Zheng, 2017). AChRs are concentrated at the mouth of postsynaptic junctional folds directly apposed to active zones of presynaptic terminals. The accumulation of AChRs under presynaptic neurotransmitter release sites may allow effective synaptic transmission (York and Zheng, 2017). The AChRs of human NMJs have only been investigated at confocal-level resolution and has not yet been analyzed using super-resolution microscopy (Jones et al., 2017).

## Age-Related Changes in the Neuromuscular Junction

The elderly, those over 65 years, suffer from a progressive loss of muscle mass (sarcopenia), strength, and function (for review see Roubenoff, 2000; Manini et al., 2013; Gonzalez-Freire et al., 2014; Willadt et al., 2018; Clark, 2019). These losses contribute to decreased mobility, increased frailty, and risk for mortality (Fried et al., 2001). Aging in humans results in a one to 1.5% loss of strength per year after the 6th decade of life (Frontera et al., 2000). In humans, approximately 10–20% of skeletal muscle mass is lost by the 7th decade of life compared to young adults (between 8 and 45 years) (Janssen et al., 2000; Vandervoort, 2002; Mitchell et al., 2012). This loss in muscle mass is likely due to motor unit loss (Lexell et al., 1988; Lexell, 1995) and subsequent atrophy of muscle fibers (Lexell et al., 1988). The loss of muscle fibers by apoptosis seems to be at an equal level between fast (Type II) and slow (Type I) muscle fiber types in humans (Lexell et al., 1983). However, there is preferential atrophy of fast fiber type compared with slow fiber type (Lexell et al., 1988; Klein et al., 2003). In mice, one study looking at muscle fiber type susceptibility in 29-month old mice demonstrated that denervation was greater in the fast extensor digitorum longus (EDL) muscle compared to the slow soleus muscle (Chai et al., 2011). However, another study using 24-month old mice showed no differences in denervation between muscle fiber types (Valdez et al., 2012). Human autopsy studies have shown that aging is associated with a gradual loss of lumbar and cervical motor neurons (Kawamura et al., 1977; Tomlinson and Irving, 1977; Mittal and Logmani, 1987; Zhang et al., 1996). Mechanisms that lead to neuronal loss with aging in humans may involve impaired trophic signaling from the nervous system, local degeneration, increased oxidative stress in motor neurons, and feedback from dysfunctional muscle (Gonzalez-Freire et al., 2014).

Early studies have demonstrated age-related changes of NMJ innervation and AChR distribution using silver and cholinesterase staining in longitudinal sections of intercostal muscles obtained from human cadavers (between 32 and 76 years old) (Oda, 1984). Another study on autopsied subjects showed a significant decrease in presynaptic membrane length and a significant increase of postsynaptic membrane length of NMJs with aging (Arizono et al., 1984). Electron microscopy showed pre- and postsynaptic changes at the ultrastructural level (Wokke et al., 1990). Aging was associated with irregularly shaped presynaptic nerve terminals and the presence of junctional folds without an opposing nerve terminal in longitudinal sections of external intercostal muscles. Postsynaptically, electron microscopy revealed increased length and branching of the postsynaptic membrane, enlarged postsynaptic area, and degeneration of junctional folds (Wokke et al., 1990). Transverse-sections prepared from autopsied or biopsied vastus lateralis muscle provided strong evidence for repeated cycles of denervation-reinnervation, in which motor unit remodeling and fiber type grouping occurred (Lindboe and Torvik, 1982; Lexell and Downham, 1991; Mosole et al., 2014; Zampieri et al., 2016). Histological analysis of aged human muscles also showed a significant accumulation of severely atrophic, smaller sized, and angulated fibers, which suggested reinnervation failure in advanced age (Mosole et al., 2014). In aged rodent muscles, rearrangement, clustering, and angulation of muscle fibers in a motor unit have also been observed (Larsson, 1995; Rowan et al., 2011).

In contrast to previous studies, a study by Jones and colleagues reported confocal micrographs of human NMJs in skeletal muscle obtained from amputation surgery and suggested conservation of synaptic structure across the lifespan (4th to 10th decades of life) (Jones et al., 2017). They observed a mild increase in axon diameter with increasing age. However, there were no age-associated changes in the innervation or fragmentation of NMJs in longitudinal sections of leg muscles, contradicting the earlier studies demonstrating age-related changes of NMJ innervation. They also report no significant change in the endplate area or muscle fiber diameter with age in the peroneus longus muscle (Jones et al., 2017). This finding is consistent with light microscopy analysis of human external intercostal muscles in which endplate size remained stable among ages (between 4 and 77 years old), and there was no sprouting of terminal axons (Wokke et al., 1990). A study by Gambino et al. (1990) in human posterior cricoarytenoid muscles (between 4 and 95 years old) demonstrated that there is no significant increase in NMJ length and the number of axonal terminal branches during adult life. It has also been reported that measures of presynaptic and postsynaptic structural relationships were maintained in the soleus muscle of the 24-month old rats despite aging-related changes (Deschenes et al., 2013). Differences between the human studies may be attributed to methodological disparities, which include (1) amputated versus post-mortem autopsied material, (2) light microscopy versus electron microscopy, (3) longitudinal-sections versus transverse-sections, and (4) different muscle types with potentially different susceptibility. Table 2 summarizes the age-dependent changes of human NMJs and how they compare to rodent NMJs.

**TABLE 2:** Age-dependent changes in human and rodent NMJs.

	**Human**	**Rodent**
**Presynaptic changes**		
Active zone density	Unknown	Decreases (Chen et al., 2012)
Active zone-specific proteins	Unknown	Selective degeneration of bassoon, piccolo and P/Q type VGCC protein level and density per synapse (Chen et al., 2012; Nishimune et al., 2012; Nishimune et al., 2016)
Synaptic vesicle density	Unknown	Decreases (Fahim and Robbins, 1982; Banker et al., 1983)
Nerve terminal branching	Increases (Oda, 1984), no change (Gambino et al., 1990)	Increases (Fahim et al., 1983; Li et al., 2011; Valdez et al., 2012)
Denervation	Increases in intercoastal (Oda, 1984) and VL muscles (Lexell and Downham, 1991; Mosole et al., 2014; Zampieri et al., 2016), no change in the peroneus longus muscle (Jones et al., 2017)	Increases in TA, plantaris, and EDL muscle (Fahim and Robbins, 1982; Deschenes et al., 2010; Valdez et al., 2010; Chai et al., 2011)
**Postsynaptic changes**		
NMJ fragmentation	No change in the peroneus longus muscle (Jones et al., 2017)	Increases in the EDL, diaphragm, soleus, sternomastoid, and TA muscles (Andonian and Fahim, 1988; Valdez et al., 2010; Li et al., 2011; Willadt et al., 2016)
Endplate area	Increases (Oda, 1984) and no change (Wokke et al., 1990) in intercostal muscles. No change in the peroneus longus muscle (Jones et al., 2017)	No change in the EDC, EDL, GM, and soleus muscles (Banker et al., 1983)
Postsynaptic folds number and regularity	Decreases (Arizono et al., 1984; Wokke et al., 1990)	Decreases (Fahim and Robbins, 1982)

*The age range for human studies is 4–95 years, and the age range for rodent studies is 21 days to 33 months (mice) and 56 days to 24 months (rats). EDC, extensor digitorum communis; EDL, extensor digitorum longus; GM, gluteus maximus; NMJ, neuromuscular junction; TA, tibialis anterior; VGCC, voltage-gated calcium channel; VL, vastus lateralis.*

Studies in mice have shown that aging leads to AChR cluster fragmentation and progressive denervation of NMJs (Andonian and Fahim, 1988; Valdez et al., 2010; Li et al., 2011; Willadt et al., 2016, 2018). NMJ denervation precedes the degradation of spinal motor neurons, suggesting a “dying back” neuropathy or distal axonopathy during the aging of mice (Valdez et al., 2010; Chai et al., 2011). We have observed that aging in mice results in a reduced number and protein levels of presynaptic active zones in innervated NMJs (Chen et al., 2012; Nishimune et al., 2016). More specifically, there is selective degeneration of active zone proteins, P/Q-type VGCC and Bassoon, at aged NMJs of 29-month-old mice compared to that of 8-month-old mice (Nishimune et al., 2016). However, Piccolo protein level at these aged NMJs remained similar to that of adult NMJs, suggesting that NMJ active zone proteins decrease selectively prior to NMJ denervation during aging in mice. Our electrophysiology study showed that the lack of Bassoon impairs P/Q-type VGCC function (Nishimune et al., 2012). Thus, the loss of active zone proteins in aged NMJs reduces synaptic vesicle release sites and calcium influx, which may lead to denervation and a loss of muscle strength. The effect of aging on presynaptic active zone proteins in humans is unknown.

Age-related functional changes of human NMJs have been analyzed using electromyography (EMG) to evaluate compound muscle action potential (CMAP) and motor unit number estimation (MUNE). Additionally, single-fiber EMG (SFEMG) has been used to analyze action potentials from individual muscle fibers and the variation in time of two action potentials of the same motor unit (jitter). The MUNE values of limb muscles decrease in older humans compared to that of the young (Galea, 1996; McNeil et al., 2005; Power et al., 2010; Hourigan et al., 2015; Piasecki et al., 2016). This decrease in MUNE values is consistent with the gradual loss of motor neurons (Kawamura et al., 1977; Tomlinson and Irving, 1977; Mittal and Logmani, 1987). In addition, MUNE values decrease in the skeletal muscles of aged mice (Sheth et al., 2018; Chugh et al., 2020). In accordance with the decrease of MUNE values and motor neuron number, the CMAP decreases in old humans compared to that of the young (Piasecki et al., 2016), and in aged mice compared to young mice (Sheth et al., 2018). SFEMG studies have found mild increases in jitter in some muscles suggesting a mild change in neurotransmission efficiency in humans between 10 and 90 years old (Bromberg and Scott, 1994).

Age-related functional changes of rodent NMJs have been studied using dissected nerve-muscle preparations, direct stimulation of motor nerves, and intracellular recordings from the muscle, which has not been done for human studies (Willadt et al., 2018). Aged mice show decreased MUNE values, as mentioned previously (Sheth et al., 2018; Chugh et al., 2020), and age-related increase of the blockade of synaptic transmission (Chugh et al., 2020). These findings seem consistent with the NMJ denervation observed in old mice. However, many studies of aged mice and rats revealed increases or no change of miniature endplate potential (mEPP) amplitude, endplate potential, and quantal content (Banker et al., 1983; Kelly and Robbins, 1983; Smith, 1988; Fahim, 1997; Willadt et al., 2016). Some of these changes may relate to the reduced Ca^2+^ clearance rates in presynaptic terminals (Smith, 1988) or increased input resistance postsynaptic membrane in some aged NMJs (Banker et al., 1983; Fahim, 1997). These age-related changes may be compensatory mechanisms for the reduced MUNE; however, they do not seem to be in accordance with the age-related increase of the blockade of synaptic transmission (Chugh et al., 2020) or the NMJ denervation observed in old mice. Furthermore, the increase of NMJ fragmentation observed in aged mice was not associated with functional impairment (Willadt et al., 2016).

Taken together, these results suggest that age-dependent changes in structure and motor unit number (denervation) occur in human NMJs, but further studies are needed to reveal the relationship between functional and structural changes related to the aging of NMJs. The sections below will discuss mouse models that reproduce key phenotypes of neuromuscular and cellular impairment seen during aging. We will also discuss potential similarities in molecular pathways between aging and neuromuscular disease because the phenotypes of human aging, and that of neuromuscular disease model mice share defects in pre- and postsynaptic NMJ structures.

## Insights From Animal Models of Accelerated Aging

Human progeroid syndromes are genetic disorders characterized by a shortened lifespan and the premature development of phenotypes, which are normally associated with advanced age (Martin, 1978). Progeroid syndromes can occur as a result of a single gene mutation (Martin, 1978; Warner and Sierra, 2003). Therefore, mouse models of progeroid syndromes have been developed to study the mechanisms and pathways involved in aging. Mouse models of accelerated aging are useful because of their short lifespan, the specific targeting of a single gene, and the phenotypic similarities with physiological aging (Harkema et al., 2016). Obtaining insights into the aging process can lead to the identification of possible targets for sarcopenia prevention. In the section below, we will discuss observations made in few mouse models of accelerated aging and compare them to neuromuscular aging phenotypes described in aged mice and elderly humans. The following mouse models were selected based on the association between the gene modifications and neuromuscular dysfunction phenotypes. The selection is not ment to cover all animal models of accelerated aging. Many reviews provide a more extensive list of progeroid mouse models associated with gene modifications resulting in additional pathologic phenotypes implicated in physiological aging, for example in the central nervous system, cardiovascular system, bones, and joints (Carrero et al., 2016; Harkema et al., 2016; Folgueras et al., 2018).

### *Zmpste24*^–/–^ Mice

Accelerated aging was observed in a mouse model with a deletion of zinc metalloproteinase (Zmpste24) required for processing of prelamin A to lamin A (Leung et al., 2001; Bergo et al., 2002). Lamin A is a component of the nuclear envelope and interacts with membrane-associated proteins to form the nuclear lamina (Aebi et al., 1986). Lamin A has been suggested to play a role in physiological aging (Scaffidi and Misteli, 2006) because a gene mutation in Lamin A (*Lmna*) causes incomplete processing of prelamin A protein and the affected children show symptoms of premature aging (De Sandre-Giovannoli et al., 2003; Eriksson et al., 2003). The *Zmpste24*^–/–^ model has a defect in prelamin A processing and is similar to the Hutchinson–Gilford progeria syndrome (Bergo et al., 2002). Muscles of *Zmpste24*^–/–^ mice exhibit characteristics of sarcopenia, including both muscle atrophy and weakness (Bergo et al., 2002; Greising et al., 2012). More specifically, the contractile capacity of muscles of the anterior leg (extensor hallucis longus, EDL and tibialis anterior) were 30–90% weaker than those of control mice (Greising et al., 2012). Consistently, hindlimb muscles (EDL, tibialis anterior, soleus, and gastrocnemius) were 32–47% smaller in the *Zmpste24*^–/–^ mice and contained significantly more collagen compared to control littermates (Greising et al., 2012). Additionally, the ankle range of motion was 70% lower in *Zmpste24*^–/–^ than control mice. Combined together, muscle atrophy, collagen accumulation, and abnormal joint mechanics contribute to poor neuromuscular performance and muscle weakness shown by *Zmpste24*^–/–^ mice (Greising et al., 2012). These phenotypes caused by decreased Zmpste24 activity are similar to those seen in aged mice and elderly humans, suggesting that defective lamin A processing plays a role in the aging process. These observations are consistent with the recent demonstration of age-related decline in the expression of lamin A/C in the synaptic region of muscles and a role of lamin A/C in preventing NMJ degeneration (Gao et al., 2020).

### *Bub1b*^H/H^ and *Bub1b*^+/GTTA^ Mice

Accelerated sarcopenia and a shortened lifespan were observed in mice with low levels of the spindle assembly checkpoint protein BubR1, including *Bub1b*^H/H^ mice with two hypomorphic alleles and *Bub1b*^+/GTTA^ mice with the human nonsense mutation 2211insGTTA (Baker et al., 2004; Wijshake et al., 2012). These mice show a decrease in muscle fiber diameter in gastrocnemius, paraspinal, and abdominal muscles, with increased intermuscular fibrosis, and impaired regenerative capacity (Baker et al., 2008; Wijshake et al., 2012). The effect of BubR1 deficiency on NMJ structure and the innervation rate remain unknown. However, BubR1 deficiency in *Bub1b*^H/H^ mice is a trigger for *Cdkn2a* locus activation, which encodes the tumor suppressors p16^Ink4a^ and p19^ARF^ (Baker et al., 2008). Both p16^Ink4a^ and p19^ARF^ are effectors of cellular senescence (Baker et al., 2008) or a state of irreversible growth arrest (Collado et al., 2007). The levels of the senescence markers, p16^Ink4a^ and p19^ARF^, were also found to be elevated in the skeletal muscle of *Bub1b*^+/GTTA^ mice (Wijshake et al., 2012). The inducible clearance of p16^Ink4a^ positive senescent cells upon administration of a drug in *Bub1b*^H/H^ mice results in attenuation of sarcopenia and premature aging (Baker et al., 2011). Furthermore, breeding *Bub1b*^H/H^ mutant mice on a p16^Ink4a^ homozygous-null background attenuated cellular senescence and premature aging in skeletal tissues, and a 25% extension in lifespan compared to *Bub1b*^H/H^ mice (Baker et al., 2008). These results, combined with the decline of BubR1 expression in aged wild-type mice (Baker et al., 2004), suggest that BubR1 may play a role in physiological aging.

### *Ercc1*^Δ^*^/–^* Mice

Genotoxic stress as a result of cumulative damage to DNA is considered a dominant mechanism underlying aging (Hoeijmakers, 2009). The excision repair cross-complementing group 1 (ERCC1) gene functions in the nucleotide excision repair pathway (Westerveld et al., 1984). Previous studies of *ERCC1* deficient patients (Jaspers et al., 2007; Ahmad et al., 2010) and *ERCC1*^–/–^ mice (Weeda et al., 1997) have indicated that a loss of *ERCC1* nuclease function causes a premature aging degenerative phenotype in several organ systems and juvenile death. The very short lifespan of *ERCC1*^–^*^/^*^–^ mice (which is less than 38 days) hinders the investigation of age-dependent neurodegeneration. Therefore, Weeda et al. (1997) also examined *ERCC1^Δ/^*^–^ mice lacking one allele and having reduced activity in the other allele due to a seven amino-acid carboxy-terminal truncation. *ERCC1*^Δ/–^ mice have a shortened life span of 6–7 months, severe locomotor deficits, and reduced ability to maintain balance (de Waard et al., 2010). Grip strength and accelerating rotarod test performances are reduced in *ERCC1*^Δ/–^ mice at 16 weeks of age. Full and partial denervation of NMJs occurs in the lumbrical muscles of *ERCC1*^Δ/–^ mice at 8 and 16 weeks. There is also a significant increase in the number of motor endplates that are innervated by more than one incoming axon collateral in *ERCC*^Δ/–^ mice (de Waard et al., 2010). This sprouting of new collateral axonal branches occurs as a compensatory mechanism to reinnervate denervated muscle fibers. These results suggest that the accumulation of DNA damage may play a role in neuronal aging and motor neuron vulnerability in aging.

### *Sod1*^–/–^ Mice

Oxidative stress is one of the leading theories on mechanisms underlying age-related muscle denervation (Fulle et al., 2004; Salmon et al., 2010). Reactive oxygen species-mediated oxidative damage to proteins, lipids, and DNA are kept in check by antioxidants under normal physiological conditions. However, excessive reactive oxygen species production can overwhelm the antioxidant defense, leading to increased oxidative damage of cellular machinery (Park, 2015). Studies on the genetic ablation of superoxide dismutase 1 (SOD1), a free radical scavenging enzyme, have shown that superoxide (O_2_^–^/HO_2_) can be toxic, and the lack of Cu/Zn SOD contributes to a deleterious phenotype in various model systems (Phillips et al., 1989; Ho et al., 1998; McFadden et al., 1999; Sanchez et al., 2005; Muller et al., 2006). Mice lacking Cu/Zn SOD1 (*Sod1*^–/–^) show phenotypes that resemble an acceleration of normal age-related sarcopenia (Muller et al., 2006) and exhibit a 30% reduction in lifespan (Elchuri et al., 2005). Hindlimb muscle mass in *Sod1*^–/–^ mice is approximately 50% lower than in age-matched wild-type mice. Skeletal muscle tissue from *Sod1^–/–^* mice show increased oxidative damage of proteins, lipids, and DNA compared to control mice. These differences are accompanied by a 40% decrease in voluntary wheel running at 6 months of age, and about 50% worse Rotarod performance in *Sod1^–/–^* mice compared to wildtype littermates (Muller et al., 2006). Interestingly, depletion of Cu/Zn SOD1 in either the motor neuron or muscle alone is not sufficient to reproduce a sarcopenic phenotype and that a loss of Cu/Zn SOD1 in both neurons and muscle is required to generate atrophy (Zhang et al., 2013; Sataranatarajan et al., 2015). Furthermore, *Sod1*^–/–^ mice display progressive NMJ denervation despite the absence of losses of spinal cord motor neurons and ventral root axons (Flood et al., 1999; Shefner et al., 1999; Jang et al., 2010; Fischer et al., 2011, 2012). Denervation and sprouting occur in these mice at 1–4 months of age, preceding muscle fiber loss that occurs between 3 and 4 months of age (Muller et al., 2006; Fischer et al., 2011, 2012). Muscle denervation and abnormalities in motor axon morphology of *Sod1^–/–^* mice are greater in the gastrocnemius and tibialis anterior muscles compared to the soleus muscle (Muller et al., 2006; Fischer et al., 2011, 2012). The gastrocnemius and tibialis anterior muscles have a higher proportion of fast muscle fibers, and the soleus muscle has a higher proportion of slow muscle fibers. Therefore, fast muscles are more vulnerable to NMJ denervation and muscle fiber loss in *Sod1*^–/–^ mice, similar to what occurs in humans aging studies (Lexell et al., 1988; Lexell, 1995). Myosin heavy chain isoform (MHC) type II (fast) muscle fibers in elderly humans are more affected than MHC isoform type I (slow) fibers during age-related NMJ denervation (Lexell et al., 1988; Lexell, 1995). Overall, these results demonstrate that Cu/Zn SOD1 is necessary for normal neuromuscular functions and suppression of age-dependent neuromuscular degeneration.

These animal models show accelerated aging with defects of the neuromuscular system including sarcopenia, axonal sprouting, and denervation like physiological aging. These mice provides opportunities to study NMJ changes, understand pathological mechanisms, and identify therapeutic targets to delay or prevent the onset of age-related neuromuscular impairments.

## Comparison With Other Diseases That Affect the NMJ

The mechanisms that form NMJs during development or degenerate NMJs in neuromuscular diseases may underlie the mechanisms of physiological aging in humans. For example, a loss in presynaptic active zones has been observed in NMJs of aged mice (Chen et al., 2012). Similarly, active zone structure is impaired in human neurological diseases, including LEMS and Pierson syndrome. LEMS patients develop autoantibodies against P/Q-type VGCCs, which is thought to cause internalization of P/Q-type VGCCs into nerve terminals (Lambert and Elmqvist, 1971; Fukunaga et al., 1982; Flink and Atchison, 2003). LEMS patients exhibit a reduced number of NMJ active zones, reduced synaptic transmission, weakened muscles, and fatigue (Lambert and Elmqvist, 1971; Fukunaga et al., 1982) similar to what is observed in aging. Interestingly, some LEMS patients develop autoantibodies specifically against the domain of P/Q-type VGCCs, where laminin β2 binds to (Takamori et al., 2000; Nishimune et al., 2004). These autoantibodies may cause a loss of interaction between the synapse organizer laminin β2 and VGCCs, which may inhibit the organization of active zones. Consistently, mice immunized with LEMS patient IgGs also show a reduced number of active zones (Fukunaga et al., 1983). Thus, the interaction between P/Q-type VGCCs and laminin β2 is essential for organizing the presynaptic active zones. Meanwhile, Pierson syndrome patients lack functional laminin β2 due to a genetic mutation and show a reduction in active zones at the NMJ, impairments in synaptic transmission including reduced mEPP frequency and amplitude, and denervation (Zenker et al., 2004; Maselli et al., 2009). These clinical characteristics of Pierson syndrome are consistent with the phenotypes of laminin β2 knockout mice (Noakes et al., 1995). Together, these observations suggest that laminin β2 plays an important role in active zone organization in humans and a potential role in the maintenance of active zones in aged NMJs.

Myasthenia gravis (MG), the most common disorder of NMJs, is caused by autoantibodies against postsynaptic membrane proteins (Conti-Fine et al., 2006; Vincent et al., 2006; Shigemoto et al., 2010). The characteristic clinical features of MG include fatigue, muscular atrophy, weakness, and ptosis (Slater et al., 2006; Phillips and Vincent, 2016). Analyses of patient sera showed that the majority of MG patients produce autoantibodies against AChRs (∼85%). Autoantibodies against muscle-specific kinase (MuSK) makeup ∼8% of MG patients and were found in 70% of MG patients who lacked autoantibodies against AChRs (Gilhus and Verschuuren, 2015; Binks et al., 2016). Furthermore, autoantibodies against LRP4 were found in 2–54% of MG patients who were negative for anti-AChR and anti-MuSK antibodies (Higuchi et al., 2011; Zhang et al., 2012; Zisimopoulou et al., 2014). Various studies using animal models that received injections of MG patient IgG have shown that these antibodies are pathogenic. AChR antibodies increase the rate of AChR internalization, and a loss of AChRs at the NMJ impairs neuromuscular transmission (Engel et al., 1977; Phillips and Vincent, 2016). The postsynaptic membrane shows a diminished response to ACh and a reduction in EPPs and mEPPs amplitudes (Loutrari et al., 1992; Ruff and Lennon, 2008). MG patients’ MuSK antibodies prevent the assembly and activation of the agrin-LRP4-MuSK complex necessary for NMJ maintenance (Huijbers et al., 2013; Otsuka et al., 2015). MuSK and LRP4-immunized mice also show impairments of neuromuscular transmission as well as a reduction in the size of motor terminals apposing AChR clusters at NMJs (Mori et al., 2012; Zhang et al., 2012; Mori et al., 2017). Transverse-sections of muscles from MuSK-immunized rabbits showed angular muscle fibers (Shigemoto et al., 2006) similar to what is seen in transverse-sections of aged muscle fibers (Larsson, 1995; Zampieri et al., 2016). LRP4-immunized mice showed significantly decreased staining areas of the presynaptic proteins, synaptophysin and bassoon, at NMJs compared to controls (Mori et al., 2017). However, the normalized bassoon staining area was unchanged compared to control mice because the synapse area is also reduced in these mice (Mori et al., 2017). It is worth noting that gene mutations in the agrin-LRP4-MuSK-Dok7-rapsyn-AChR pathway, including the genes *AGRN, MUSK, DOK7, RAPSN*, cause congenital myasthenic syndromes (CMSs). CMSs are characterized by weakness and fatigue, similar to MG (Rothbart, 1937). However, CMS is not a degenerative disorder like aging but a developmental disorder, which has been reviewed previously (Engel et al., 2010, 2015; Webster, 2018). In aged mice, the agrin-LRP4-MuSK-AChR signaling pathway is implicated in aging-associated NMJ deficits (Zhao et al., 2018). LRP4 protein levels are decreased in aged muscles and restoring the levels of LRP4 by transgenic expression or stabilization with sarcoglycan alpha maintained NMJ innervation, alleviated AChR fragmentation, and improved synaptic transmission (Zhao et al., 2018). Furthermore, viral-mediated upregulation of *DOK7* in aged mouse muscle significantly enhanced motor function, muscle strength, NMJ innervation, and compound muscle action potential amplitudes (Ueta et al., 2020). Thus, studying the role of the agrin-LRP4-MuSK-Dok7-rapsyn-AChR signaling pathway in aging has identified a potential therapeutic target for alleviating NMJ decline in aging. This elucidation was helped by the prior knowledge demonstrating the importance of this signaling pathway for NMJ development, maintenance, and degeneration in MG (Li et al., 2018).

Amyotrophic lateral sclerosis (ALS) is a neurodegenerative disorder characterized by a gradual loss of motor neurons that leads to paralysis and death (Brown, 1995). The first gene mutations associated with familial ALS were identified in the *SOD1* gene (Rosen et al., 1993). These findings led to the development of the transgenic mouse model for ALS expressing the human SOD1 gene with glycine 93 to alanine (G93A) mutation identified in patients (SOD1^G93A^ mice) (Gurney et al., 1994). SOD1^G93A^ mice exhibit progressive denervation, motor neuron loss, muscle weakness, and paralysis similar to ALS patients (Gurney et al., 1994; Chiu et al., 1995; Tu et al., 1996; Wong and Martin, 2010; Rocha et al., 2013; Dobrowolny et al., 2018). NMJ denervation is observed as early as 47 days of age in these mice (Kennel et al., 1996; Fischer et al., 2004) and precedes both motor neuron loss (Gurney et al., 1994; Dadon-Nachum et al., 2011) and muscle atrophy (Brooks et al., 2004; Marcuzzo et al., 2011). These characteristics are similar to those observed in the rodent models of aging (Chai et al., 2011; Valdez et al., 2012) as well as in *SOD1^–/–^* mouse models of accelerated aging (Shi et al., 2014). In addition, SOD1^G93A^ mice display preferential denervation of fast-twitch muscles (Atkin et al., 2005; Hegedus et al., 2007, 2008). It has been suggested that both fast type muscle and motor units are preferentially vulnerable to the disease process in ALS patients (Dengler et al., 1990; Sanjak et al., 2001). This increased susceptibility to NMJ denervation in fast type muscles has also been demonstrated in aged mice (Chai et al., 2011; Valdez et al., 2012) and *SOD1^–/–^* mouse models of accelerated aging (Fischer et al., 2011, 2012).

Identifying similarities between aging and phenotypes of neurodegenerative diseases may suggest common molecular pathways that underlie aging-related degeneration of NMJs. Furthermore, understanding the molecular basis of NMJ dysfunction in animal models is essential for translating research to study aging in humans and search for candidate targets for therapeutic intervention of sarcopenia and frailty. For example, a mechanism of peripheral neuropathy elucidated from aged mice may provide a novel therapeutic approach for age-related degeneration observed in the elderly (Yuan et al., 2018). The previous reviews have discussed the possible functional significance associated with age-related changes in NMJ structure, which include denervation, fragmentation, axonal branching, decreased synaptic vesicles, and altered postsynaptic folding (Willadt et al., 2016; Li et al., 2018). Importantly, studying NMJ dysfunction is essential for understanding musculoskeletal impairment during aging.

## Interventions to Improve Age-Related NMJ Dysfunction in Humans

Exercise and caloric restriction attenuate age-related declines in the mammalian neuromuscular system and are potential interventions to delay the onset of age-related NMJ dysfunction and sarcopenia (Cadore et al., 2014; Most et al., 2017; Lopez et al., 2018; Lavin et al., 2019). Aging leads to a loss of motor units, as described in the previous section titled “age-related changes in the NMJ.” In humans, a study on master athletes indicates that high levels of life-long physical activity may ameliorate this loss of functional motor units in the tibialis anterior muscle into the 7th decade of life compared to age-matched controls (Power et al., 2010). Unlike the tibialis anterior leg muscle, the estimated number of functional motor units in the biceps brachii arm muscle of masters runners was lower in old age compared with young adults (Power et al., 2012). These findings indicate that high levels of chronic activation is necessary for delaying the age-related loss of motor units, and the beneficial effect is specific to the muscle exposed to the long term high levels of physical activity (Power et al., 2012). The number of functional motor units in a human muscle group can be estimated using a minimally invasive electrophysiological technique (decomposition-enhanced spike-triggered averaging) (McComas et al., 1971). This technique can also provide indirect evidence of collateral reinnervation using the peak amplitude of the mean surface-detected motor unit potential (Power et al., 2016). World champion master runners in their ninth decade of life had a greater number of remaining motor units and reduced collateral reinnervation compared to age-matched non-athletes (Power et al., 2016). Furthermore, master athletes had a 14% greater amount of excitable muscle mass and better neuromuscular transmission stability or preterminal axon stability, as indicated by lower jitter values, compared with age-matched non-athletes (Power et al., 2016). Additionally, biopsies from seniors physically active at high-level (65–79 years old) revealed a significantly higher percentage of slow-type myofibers and increased muscle fiber-type groupings, suggesting that long-term cycles of denervation/reinnervation have occurred (Mosole et al., 2014). People aged 70 years or older who reported walking 4–7 days per week showed a reduced risk of lower-body mobility impairments compared to elderly who reported walking fewer days per week (Clark, 1996). Moderate to high levels of physical activity in aged adults was associated with a significant increase in life expectancy and disability-free years before death compared to aged adults who reported low physical activity (Ferrucci et al., 1999). Starting exercise training in old age can also lead to significant health improvements. Nine weeks of training improved muscle strength and recovered myofiber atrophy in 70-year-old subjects compared to pre-training (Zampieri et al., 2016). Together, these results demonstrate that exercise maintains neuromuscular stability and ameliorates the loss of motor units associated with aging well into the later decades of human life.

In rodents, endurance exercise has a beneficial effect on NMJs in the aging muscle. Running wheel and treadmill running has been shown to reduce post-synaptic AChR fragmentation related to rodent aging, and help maintain NMJ innervation and endplate area (Andonian and Fahim, 1988; Fahim, 1997; Valdez et al., 2010; Deschenes et al., 2011; Cheng et al., 2013). Presynaptically, exercise has been shown to maintain active zone protein content. Active zone protein levels in NMJs are reduced during aging, but resistance training ameliorated the loss of Bassoon at NMJs of 24-month-old aged rats to young adult levels (Nishimune et al., 2012). This finding is consistent with endurance training improving NMJ function in aged mice, which was revealed using electrophysiology (Fahim, 1997). To our knowledge, no other studies investigated active zone proteins at NMJs combined with exercise training. More work is needed to understand the molecular mechanisms by which exercise modulates active zones and active zone proteins during aging.

Caloric restriction is a long-term dietary intervention in which caloric intake is reduced, but malnutrition is avoided (Anderson and Weindruch, 2012). The first description of the possible benefits and mechanisms of caloric restriction in aging has been demonstrated through the studies of rodent and invertebrate species (Fontana et al., 2010; Anderson and Weindruch, 2012). The caloric restriction prolonged the lifespan of rodents in an inverse linear manner when caloric intake is reduced at 10–30% compared to *ad libitum* intake of a standard diet (Weindruch et al., 1986; Anderson and Weindruch, 2010). Furthermore, a 40% reduction in caloric intake in mice from 4 to 24 months of age led to the maintenance of NMJs in the tibialis anterior muscle compared to control mice fed a standard diet *ad libitum* (Valdez et al., 2010). The frequencies of AChR fragmentation and NMJ denervation were significantly lower in these calorically restricted mice than in control aged mice indicating an attenuation of the age-related changes in the NMJ structure (Valdez et al., 2010). The use of the caloric restriction mimetic, resveratrol had a beneficial effect on NMJs and preserving muscle fiber morphology in the EDL muscle of 2-year-old mice (Stockinger et al., 2017). The beneficial effect of caloric restriction on health has also been reported in non-human primates (Roth et al., 2000). In rhesus monkeys, caloric restriction of 30% reduced intake improves survival and delays the onset of sarcopenia and age-associated diseases (McKiernan et al., 2011). Analysis of vastus lateralis muscle biopsies showed that caloric restriction attenuated age-related changes in the proportion of Type II muscle fibers and fiber transverse-sectional area (McKiernan et al., 2011). Epidemiological studies show evidence that caloric restriction without malnutrition may also have health-promoting effects in humans. A large population of centenarians is found on the island of Okinawa, where individuals reportedly eat fewer calories on average than people in mainland Japan (Chan et al., 1997). Randomized controlled trials of caloric restriction in the United States show a decline in markers of oxidative stress and DNA-damage after 6 months compared to controls on a weight-maintenance diet (Heilbronn et al., 2006; Civitarese et al., 2007). Skeletal muscle biopsies collected from the trial participants showed that caloric restriction induces mitochondrial biogenesis and decreases superoxide dismutase activity, which in turn lowers oxidative stress (Civitarese et al., 2007). A caloric restriction trial in healthy, adult humans demonstrated that a 15% caloric restriction over 2-years resulted in beneficial metabolic adaption and a reduction in biomarkers associated with aging compared to an *ad libitum* control group (Redman et al., 2018). Studies in *Caenorhabditis elegans*, *Drosophila*, and yeast suggest a possible link between the TOR (Target of Rapamycin) pathway and caloric restriction (Kapahi et al., 2004; Kaeberlein et al., 2005; Hansen et al., 2007). The potential role of mTOR signaling in the aging process of these model organisms in addition to mammals has been reviewed previously (Saxton and Sabatini, 2017; Papadopoli et al., 2019). In mice, a sirtuin, Sirt1 has been shown to regulate aging and longevity. Brain specific overexpression of *Sirt1* in mice protected against the age-related decline in skeletal muscle and resulted in more youthful-appearing NMJs (Ghosh and Zhou, 2015; Snyder-Warwick et al., 2018). The mechanisms linking Sirt1 from the hypothalamus to skeletal muscle in mice and the involvement of SIRT1 in humans remains to be elucidated. Aside from caloric restriction, there is evidence for a beneficial effect of specific nutrients and dietary supplements on the aged neuromuscular system in humans. A recent review discussed potential mechanisms of promoting healthy neuromuscular aging by β-hydroxy-β-methylbutyrate, creatine, dietary phospholipids, omega-3 fatty acids, and vitamin D (Kougias et al., 2018). Additional clinical research investigating optimal dosages and durations of caloric restriction and nutritional intervention is needed to determine whether these effects can attenuate the aging process.

Examining the pathological mechanisms of neurodegenerative diseases with phenotypes similar to aging will help evaluate the molecular changes that underlie the structural and functional changes seen during aging. These include changes in active zone number and composition, NMJ innervation, and muscle mass. Also, mouse models of accelerated aging add value by identifying new pathways involved in aging. Importantly, the conserved beneficial effect in rodents, non-human primates, and humans supports the importance of caloric restriction and exercise for improving physical function in the elderly. Taken together, these strategies are likely to contribute to the development of interventions for preventing age-related neuromuscular dysfunction, sarcopenia, and frailty.

## Author Contributions

YB and HN contributed to the conception and writing of this review manuscript. Both authors contributed to the article and approved the submitted version.

## Conflict of Interest

The authors declare that the research was conducted in the absence of any commercial or financial relationships that could be construed as a potential conflict of interest.

## Abbreviations

ACh: acetylcholine
AChR: acetylcholine receptors
ALS: amyotrophic lateral sclerosis
CMS: congenital myasthenic syndromes
dSTORM: direct stochastic optical reconstruction microscopy
ERCC1: excision repair cross-complementing group 1
LEMS: Lambert-Eaton myasthenia syndrome
LRP4: low-density lipoprotein receptor-related protein 4
MuSK: muscle-specific kinase
MG: myasthenia gravis
MHC: myosin heavy chain isoform
mTOR: mammalian/mechanistic Target of Rapamycin
NMJ: neuromuscular junction
SOD1: superoxide dismutase 1
STED: stimulated emission depletion
SNAP25: synaptosomal associated protein-25
SNARE: soluble *N*-ethylmaleimide -sensitive factor activating protein receptor
VGCC: voltage-gated calcium channels.
